# Incidence, risk factors, and clinical outcomes of acute kidney injury associated with acute pyelonephritis in patients attending a tertiary care referral center

**DOI:** 10.1080/0886022X.2019.1591995

**Published:** 2019-04-03

**Authors:** Dae-Hong Jeon, Ha Nee Jang, Hyun Seop Cho, Tae Won Lee, Eunjin Bae, Se-Ho Chang, Dong Jun Park

**Affiliations:** a Department of Internal Medicine, Gyeongsang National University Hospital, Jinju, Republic of Korea;; b Department of Internal Medicine, Changwon Gyeongsang National University Hospital, Changwon, Republic of Korea;; c Department of Internal Medicine, College of Medicine, Gyeongsang National University, Jinju, Republic of Korea;; d Institute of Health Science, Gyeongsang National University, Jinju, Republic of Korea

**Keywords:** Acute kidney injury, pyelonephritis, risk factor

## Abstract

Acute kidney injury (AKI) associated with acute pyelonephritis (APN) rarely has been reported. The aim of this study was to evaluate the incidence and risk factors of AKI associated with APN. We retrospectively reviewed the medical records of 403 patients over 18-year old age hospitalized for APN management from October 2009 to September 2014 in tertiary care referral center. Demographic data, clinical symptoms and signs, and laboratory findings were gathered from the medical records and analyzed. The mean age of patients was 57 years and APN commonly occurred in female (87.6%). AKI occurred in 253 patients (62.8%). As per the RIFLE classification, renal injury was graded as ‘Risk’ (62.1%), ‘Injury’ (26.5%), and ‘Failure’ (11.4%). AKI patients were more likely a male gender and had complicated APN. The AKI group had a significantly higher tendency to present with shock. The prevalence of underlying chronic kidney disease (CKD) was significantly higher in the AKI group. There was no difference in mortality between the AKI and non-AKI groups. Multivariate analysis revealed that age over 65 (OR 1.93, 95% CI 1.18–3.13, *p*= .008), complicated (OR 2.13, 95% CI 1.35–3.34, *p*= .001) and bilateral APN (OR 1.71, 95% CI 1.01–2.88, *p*= .045), and initial shock (OR 2.44, 95% CI 1.05–5.71, *p*= .039) were independent risk factors for the occurrence of AKI in patients with APN. Physicians should attempt to prevent, detect, and manage AKI associated with APN in patients with above conditions.

## Introduction

Acute pyelonephritis (APN) is the most common bacterial infection involving the upper urinary tract system, including the renal parenchyma and pelvis. APN accounts for approximately 250,000 office visits and 200,000 hospital admissions each year in the United States [1], and the estimated annual incidence per 100,000 people is 35.7 cases in South Korea [2]. Clinical history and physical examination are the most useful components of diagnosis. The clinical presentation of APN includes lower urinary tract symptoms such as frequency, urgency, and dysuria accompanied by fever, upper gastrointestinal symptoms, headache, and flank pain [3,4]. Flank pain is almost universal in patients with APN, and its absence is suggestive of an alternative diagnosis requiring additional diagnostic tools for confirmation. Urine cultures are positive in 90% of patients with APN, and cultures should be performed prior to antibiotic therapy. Blood culture should be reserved for patients with an uncertain diagnosis [3]. Complications include renal abscess, septic shock, and renal impairment, including acute renal failure (ARF).

Acute kidney injury (AKI) is an important clinical problem in patients with infectious diseases. According to the applied criteria, the incidence of AKI ranges from 5 to 51% in patients with sepsis [5–9]. Additionally, AKI is as an independent poor prognostic factor in sepsis. Many studies have shown that several factors contribute to the occurrence and outcome of AKI in septic patients [9–12]. The association between APN and ARF, severe forms of AKI, are reported as only sporadic case series [13–16] or only one category of the clinical characteristics of APN [17–19]. In 2004, the Acute Dialysis Quality Initiative (ADQI) group developed a consensus definition of AKI, with the introduction of the Risk, Injury, Failure, Loss, and End-stage renal disease (RIFLE) classification, which has been validated in terms of determining the incidence of AKI [20]. However, no study has applied this RIFLE classification to evaluate the incidence, risk factors, or clinical outcomes of AKI associated with APN. Therefore, in this study, we explored the incidence, risk factors, and clinical outcomes of AKI associated with APN by applying the RIFLE classification, the first international interdisciplinary consensus classification for the diagnosis of AKI.

## Materials and methods

### The selection of the patients

We retrospectively reviewed the medical records of 403 hospitalized patients over 18 years of age who were treated for APN at Gyeongsang National University Hospital from October 2009 to October 2014. All patients had documented medical histories and underwent review. Demographic and clinical characteristics, laboratory findings, and comorbidities were gathered from the medical records and analyzed. To evaluate the incidence, risk factors, and clinical outcomes of AKI in patients with APN, we divided the patients into two groups: non-AKI and AKI groups. We also divided the patients into two groups, non-CKD and CKD groups, to evaluate AKI occurrence and severity according to RIFLE classification and clinical outcomes.

### Definitions

Medical history and physical examination were the most useful tools for the diagnosis of APN characterized by pain when passing urine, chills, flank pain, nausea, vomiting, fever (≥38.5 °C) and CVA tenderness. A positive urinalysis such as pyuria on urine microscopy (WBC ≥ 5/HPF) and positive nitrite test and/or positive urine culture (≥100,000 CFU/mL) of a mid-stream specimen confirms the diagnosis in patients with a compatible history and physical examination. Abdominal computed tomography (CT) was also helpful in diagnosing complicated APN such as obstructed kidney, high risk patients including diabetics, elderly, and immunocompromised persons.

Complicated APN was defined when the patient had at least one risk factor among diabetes mellitus (DM), pregnancy, urologic anatomical abnormalities, urinary tract stones, malignancy, or an immunocompromised state. Patients with none of the medical problems described above were defined as uncomplicated APN. Shock was defined as a systolic blood pressure ≤90 mmHg. Bacteremia was defined as in the case of positive blood culture. Bilateral APN was defined as follows; flank pain and/or CVA tenderness existed in both sides, typical CT findings were found on both kidney, and APN occurred in single kidney.

Chronic kidney disease (CKD) was defined as an estimated glomerular filtration rate (eGFR)<60 mL/min/1.73 m^2^ using the Modification of Diet in Renal Disease study (MDRD) formula [1.86×(plasma creatinine) – 1.154×(age) – 0.203)]×(0.74 if female)×(1.210 if black). The most recent past plasma creatinine level before hospitalization was used as baseline value if the previous renal function was known.

The method of creatinine formation was Jaffe one. RIFLE classification has usually been used to evaluate AKI incidence in our institution because RIFLE classification can easily be applied when the baseline serum creatinine is known and has been largely validated in terms of determining the incidence of AKI. AKI was defined and categorized according to the RIFLE classification [20]. We only used the serum creatinine level and eGFR to establish the RIFLE category because the urine output of APN patients was not routinely recorded. If the baseline creatinine values were unknown, the serum creatinine was estimated using the MDRD formula and assuming eGFR = 75 mL/min/1.73 m^2^ as the normal value. To evaluate the incidence, risk factors, and clinical outcomes of AKI associated with APN, we divided the patients into AKI and non-AKI groups. We also compared above categories with dividing patients into CKD and non-CKD groups. We evaluated the severity of AKI categorized using RIFLE in CKD versus non-CKD groups.

The study protocol was approved by the Institutional Review Board at Gyeongsang National University Hospital (IRB No.: 2016-01-014).

### Statistical analysis

All measurements were expressed as the mean ± standard deviation (SD). We present the data as absolute numbers and percentage with 95% confidence interval (CI). We used Pearson’s chi-square test to analyze qualitative differences. The parametric Student’s *t*-test was used to compare means of samples of similar variance. A multivariate logistic regression analysis was used to identify significant risk factors for the occurrence of AKI among those identified in univariate analysis. Statistical analysis was performed using a commercially available statistical software package (V.21.0; SPSS, Chicago, IL). A *p* value of less than .05 was considered statistically significant in all analyses.

## Results

### The incidence of AKI and risk factors in patients with APN

Table 1 lists demographic and clinical data, comorbidities, and laboratory findings for all patients. The incidence of AKI with APN was 62.8% (253/403). As per RIFLE classification, patients with AKI were categorized as ‘Risk’ (157 patients; 62.1%), ‘Injury’ (67 patients; 26.5%), and ‘Failure’ (29 patients; 11.5%). The mean age of the AKI group was significantly higher than that of the non-AKI group and the rate of patients who were older than 65 years was also significantly higher in the AKI group. Patients with AKI were more likely to have a male gender (*p*= .008) and complicated APN (*p*= .000). There were no significant differences in symptoms of febrile sensation, nausea, vomiting, and urinary symptoms. However, flank pain was less common in the AKI group (*p*= .000). The AKI group had a significantly higher tendency to present with shock (*p*= .024), and the duration of admission was significantly longer in the AKI group (*p*= .000). The prevalence of underlying CKD was also significantly higher in the AKI group (*p*= .005). Higher WBC and CRP levels and lower hemoglobin, platelet, and albumin were common in AKI patients (*p*< .001, *p*< .001, *p*= .013, *p*< .001, and *p*< .001, respectively). Bacteremia was more common in the AKI group (*p*=.003) compared with the non-AKI group. Multivariate analysis revealed that age over 65 (OR 1.93, 95% CI 1.18–3.13, *p*= .008), complicated (OR 2.13, 95% CI 1.35–3.34, *p*= .001) and bilateral APN (OR 1.71, 95% CI 1.01–2.88, *p*= .045), and initial shock (OR 2.44, 95% CI 1.05–5.71, *p*= .039) were independent risk factors for the occurrence of AKI in patients with APN (Table 2).

**Table 1.** Clinical and laboratory data of the AKI group and non-AKI group.

Characteristics	Total (*n* = 403)	Non-AKI (*n* = 150)	AKI (*n* = 253)	*p* Value
Age	57.26 ± 17.81	50.04 ± 17.79	61.53 ± 16.42	<.001
>65 years (%)	164 (40.7%)	61 (24.4%)	103 (75.6%)	<.001
Sex				.008
Male (%)	50 (12.4%)	10 (6.7%)	40 (15.8%)	
Female (%)	353 (87.6%)	140 (93.3%)	213 (84.2%)	
Symptoms				
Febrile sensation (%)	356 (88.6%)	134 (89.3%)	222 (87.7%)	.749
Nausea/vomiting (%)	86 (21.3%)	29 (19.3%)	57 (22.5%)	.530
Flank pain (%)	117 (29.0%)	60 (40.0%)	57 (22.5%)	<.001
Urinary symptoms (%)	177 (43.9%)	67 (44.7%)	110 (43.5%)	.836
Complicated (%)	187 (46.4%)	49 (32.7%)	138 (54.5%)	<.001
DM (%)	102 (25.3%)	22 (14.7%)	80 (31.6%)	<.001
Anatomical abnormalities (%)	66 (16.4%)	15 (10.0%)	51 (20.2%)	.008
Urinary stone (%)	33 (8.2%)	7 (4.7%)	26 (10.3%)	.059
Pregnancy (%)	7 (1.7%)	4 (2.7%)	3 (1.2%)	.432
Immunosuppressant use (%)	4 (1.0%)	1 (0.7%)	3 (1.2%)	1.000
Malignancy (%)	31 (7.7%)	8 (5.3%)	23 (9.1%)	.245
Shock (%)	40 (9.9%)	8 (5.3%)	32 (12.6%)	.024
Bilateral APN (%)	113 (28.0%)	29 (19.3%)	84 (33.2%)	.003
Admission duration (day)	8.6 ± 5.5	6.2 ± 2.2	10.1 ± 6.3	<.001
CKD (%)	30 (7.4%)	4 (2.7%)	26 (10.3%)	.005
Death (%)	4 (0.99%)	2 (1.3%)	2 (0.79%)	.631
WBC (×10^3^/µL)	13.15 ± 6.67	11.55 ± 4.14	14.10 ± 7.64	<.001
Hemoglobin (g/dL)	11.57 ± 1.55	11.97 ± 1.37	11.33 ± 1.61	.013
Platelet (×10^3^/mm^3^)	206.18 ± 87.94	225.09 ± 79.89	194.96 ± 90.69	.000
ESR (mm/h)	54.82 ± 27.75	52.16 ± 24.00	56.45 ± 29.75	.138
CRP (mg/L)	143.30 ± 91.78	115.24 ± 84.47	159.94 ± 92.04	<.001
Albumin (g/dL)	3.47 ± 0.61	3.71 ± 0.50	3.33 ± 0.63	.000
Phosphorus (mg/dL)	2.82 ± 0.94	2.82 ± 0.83	2.82 ± 1.00	.989
Na (mmol/L)	136.10 ± 3.90	136.82 ± 3.04	135.66 ± 4.27	.002
K (mmol/L)	3.86 ± 0.53	3.79 ± 0.43	3.91 ± 0.57	.023
BUN(mg/dL)	20.30 ± 14.62	13.00 ± 6.53	24.63 ± 16.29	<.001
Cr (mg/dL)	1.18 ± 1.06	0.74 ± 0.28	1.43 ± 1.25	<.001
Bacteremia (%)	145 (36.0%)	40 (26.7%)	105 (41.5%)	<.001

WBC: white blood cell; ESR: erythrocyte sedimentation ratio; CRP: C-reactive protein; BUN: blood urea nitrogen; Cr: creatinine; DM: diabetes mellitus; CKD: chronic kidney disease.

**Table 2.** Independent risk factors for the development of AKI.

Characteristics	Univariate analysis			Multivariate analysis		
	*p* Value	OR	95% CI	*p* Value	OR	95% CI
Age > 65 years	<.001	2.643	1.706–4.096	.008	1.925	1.184–3.130
Sex (female)	.008	0.380	0.184–0.785	.109	0.527	0.240–1.154
Flank pain	<.001	0.436	0.281–0.677	.072	0.640	0.394–1.040
Complicated	<.001	2.473	1.623–3.770	.001	2.127	1.353–3.341
Shock	.021	2.570	1.151–5.737	.039	2.443	1.045–5.709
Bilateral APN	.003	2.074	1.280–3.359	.045	1.707	1.013–2.876
CKD	.009	4.181	1.430–12.225	.120	2.438	0.794–7.493
Bacteremia	.003	1.951	1.257–3.029	.068	1.579	0.968–2.577

OR: odds ratio; CI: confidence interval; CKD: chronic kidney disease.

### AKI incidence and severity between non-CKD and CKD groups

**Figure 1.**
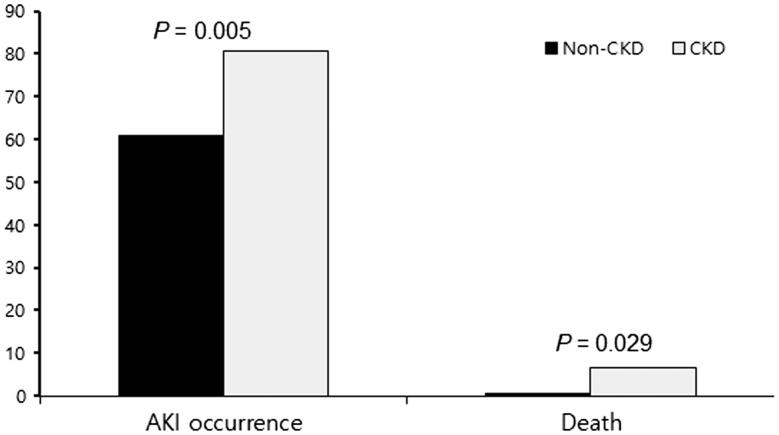
Percentage of AKI occurrence and death according to presence of CKD.

**Figure 2.**
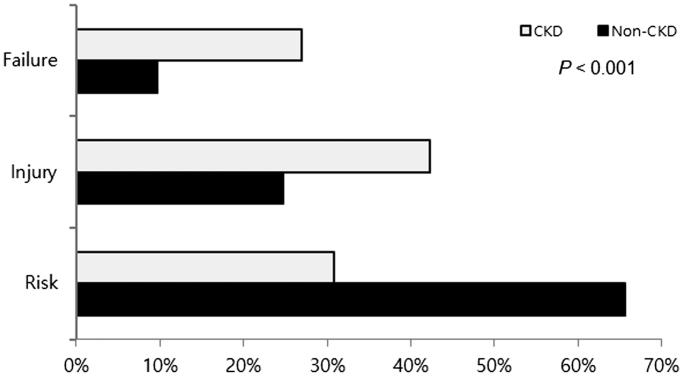
The comparison of AKI according to the RIFLE category in patients with CKD and non-CKD.

Table 3 lists demographic and clinical data, comorbidities, and laboratory findings in non-CKD and CKD patients. The mean age of the CKD group was significantly higher than that of the non-CKD group, and the rate of patients who were older than 65 years was also significantly higher in the CKD group. Complicated APN was significantly higher in the CKD group (*p*= .023). Flank pain was less common in the CKD group (*p*=.02). The duration of admission was significantly longer in the CKD group (*p*< .001). Lower hemoglobin and albumin were observed in patients with CKD (*p*< .001 and *p*< .001, respectively). The incidence and severity of AKI based on the RIFLE classification were significantly higher in CKD patients compared with non-CKD patients (Figures 1 and 2).

**Table 3.** Clinical and laboratory data in patient with non-CKD and CKD.

Characteristics	Total (*n* = 403)	Non-CKD (*n* = 373)	CKD (*n* = 30)	*p* Value
Age	57.26 ± 17.81	56.39 ± 18.05	68.03 ± 9.10	<.001
>65 years (%)	164 (40.7%)	144 (38.6%)	20 (66.7%)	.003
Sex				.079
Male (%)	50 (12.4%)	43 (11.5%)	7 (23.3%)	
Female (%)	353 (87.6%)	330 (88.5%)	23 (76.7%)	
Symptoms				
Febrile sensation (%)	356 (88.6%)	331 (88.7%)	25 (83.3%)	.374
Nausea/vomiting (%)	86 (21.3%)	80 (21.4%)	6 (20.0%)	1.000
Flank pain (%)	117 (29.0%)	114 (30.6%)	3 (10.0%)	.020
Urinary symptoms (%)	177 (43.9%)	169 (45.3%)	8 (26.7%)	.056
Complicated (%)	187 (46.4%)	167 (44.8%)	20 (66.7%)	.023
DM (%)	102 (25.3%)	82 (22.0%)	20 (66.7%)	<.001
Anatomical abnormalities (%)	66 (16.4%)	59 (15.8%)	7 (23.3%)	.304
Urinary stone (%)	33 (8.2%)	31 (8.3%)	2 (6.7%)	1.000
Pregnancy (%)	7 (1.7%)	7 (1.9%)	0 (0.0%)	1.000
Immunosuppressant use (%)	4 (1.0%)	4 (1.1%)	0 (0.0%)	1.000
Malignancy (%)	31 (7.7%)	26 (7.0%)	5 (16.7%)	.069
Shock (%)	40 (9.9%)	35 (9.4%)	5 (16.7%)	.203
Bilateral APN (%)	113 (28.0%)	102 (27.3%)	11 (36.7%)	.293
Admission duration (days)	8.6 ± 5.5	8.3 ± 4.9	13.1 ± 9.3	<.001
AKI (%)	253 (62.8%)	227 (60.9%)	26 (86.7%)	.005
Death (%)	4 (0.99%)	2 (0.54%)	2 (6.7%)	.029
WBC (×10^3^/µL)	13.15 ± 6.67	13.08 ± 6.38	14.10 ± 9.65	.574
Hemoglobin (g/dL)	11.57 ± 1.55	11.70 ± 1.45	9.84 ± 1.80	<.001
Platelet (×10^3^/mm^3^)	206.18 ± 87.94	208.43 ± 87.08	178.20 ± 95.10	.070
ESR (mm/h)	54.82 ± 27.75	53.75 ± 27.22	67.21 ± 31.21	.014
CRP (mg/L)	143.30 ± 91.78	141.13 ± 90.21	170.28 ± 107.50	.094
Albumin (g/dL)	3.47 ± 0.61	3.52 ± 0.59	2.93 ± 0.58	<.001
Phosphorus (mg/dL)	2.82 ± 0.94	2.75 ± 0.84	3.60 ± 1.55	.006
Na (mmol/L)	136.10 ± 3.90	136.21 ± 3.73	134.72 ± 5.46	.155
K (mmol/L)	3.86 ± 0.53	3.82 ± 0.49	4.38 ± 0.70	<.001
BUN(mg/dL)	20.30 ± 14.62	18.08 ± 10.71	47.91 ± 25.31	<.001
Cr (mg/dL)	1.18 ± 1.06	0.99 ± 0.49	3.55 ± 2.49	<.001
Bacteremia (%)	145 (36.0%)	133 (35.7%)	12 (40.0%)	.694

WBC: white blood cell; ESR: erythrocyte sedimentation ratio; CRP: C-reactive protein; BUN: blood urea nitrogen; Cr: creatinine; DM: diabetes mellitus; CKD: chronic kidney disease.

### Clinical outcomes of AKI

Most patients recovered from APN and AKI with general care, such as fluid replacement and adequate antibiotic therapy, excluding four patients who died (0.99%). No patients started renal replacement therapy and there was no case of progression to CKD in non-CKD groups. Hospital stay was prolonged in AKI patients (*p*=.003). However, there were no significant differences in mortality between non-AKI and AKI groups (Table 1). Two cases of death occurred in the CKD group, and one case was accompanied with AKI. Mortality was significantly higher in the CKD group compared with the non-CKD group (*p*=.029) (Table 3, Figure 2). There was no case of end-stage renal disease requiring maintenance hemodialysis at the 3-month follow-up in the CKD group. The major cause of death was uncontrolled infection.

## Discussion

In our study, the incidence of AKI in patients with APN was 61.2%. According to the RIFLE classification, AKI was associated with renal ‘Risk’ (62.1%), ‘Injury’ (26.5%), and ‘Failure (11.5%). Independent risk factors of AKI associated with APN in the multivariate analysis were older age, initial shock, bilateral involvement of the kidney, and complicated cases, whereas sex, CKD, the presence of flank pain, and bacteremia were not associated with AKI, although these variables were significant in the univariate analysis. In patients with underlying CKD, AKI was more common and had a poorer prognosis in terms of severity and survival.

Few studies have reported AKI associated with APN according to the RIFLE classification [18,19], and these studies did not explore incidence, risk factors, and clinical outcomes of AKI. One study used clinical presentation and one study used outcome as only one category in the clinical characteristics of ipsilateral and bilateral APN: they reported incidences of 32.2% and 21%, respectively. A recent study explored risk factors for the development of AKI in patients with urinary tract infections (UTIs) [21] it reported a 12.3% incidence of AKI and concluded that risk factors associated with AKI included old age, DM, upper UTI, and underlying CKD. Its lower reported AKI incidence may have resulted from the enrollment of lower UTI patients (67%) and application of different AKI criteria. AKI was diagnosed by a decrease in GFR more than 50% or doubling of serum creatinine compared to that at baseline according to the RIFLE GFR criteria in that study [21]. Our study may be superior because we enrolled many patients with APN (291 versus 403). Thus, the higher incidence of AKI in our study may be associated with the characteristic of the disease itself that directly invades the kidney, more inclusion of complicated APN patients (46.4%), and more severe patients hospitalized to our institution with distinct characteristics of university hospital.

AKI is a common complication of sepsis and septic shock. UTI is a common cause of sepsis, may decrease renal function. Sepsis is known to be an important risk factor of AKI in several infectious diseases [7,10,22]. Previous studies have shown that hypovolaemia, hypotension, shock, the use of nephrotoxic drugs, and urinary tract obstruction are important AKI risk factors in UTI patients [15,23]. A recent study also found that older age, diabetes, upper UTI, and CKD were independent associated risk factors for developing AKI in patients with UTI [21]. In our study, complicated APN including DM, urinary tract obstruction, and initial shock were strongly associated with AKI in patients with APN. One report also showed that severe inflammation was correlated with the occurrence of AKI in patients with APN [17]. Severe leukocytosis and higher CRP, as well as lower albumin levels, were common in AKI patients compared with non-AKI patients.

CKD is considered an important risk factor for the development of AKI in several infectious diseases [21,24,25]. The frequency and severity of AKI according to the RIFLE classification were significantly greater in patients with CKD compared to patients without CKD. CKD was also associated with increased mortality in patients with AKI [24,25]. We also found that more frequent and severe AKI, and higher mortality, were common in patients with CKD and AKI in spite of few enrollments of CKD patients. Another concern of AKI prognosis without underlying CKD is progression to CKD and aggravation of CKD after AKI. According to some studies, rates to new onset of CKD after AKI range from 7 to 20% over a follow-up of 1–3 years [26–28]. However, we did not observe CKD progression after AKI in our study. These characteristics of CKD patients should not be generalized because of small enrollment. This was one of another limitation of our study.

Flank pain is almost universal in patients with APN; its absence may support an alternative diagnosis and require another diagnostic tool, leading to late diagnosis and treatment and a poor prognosis. Older patients may not present with this symptom for several reasons including hypoesthesia, mental deterioration, and poor communication with medical staff. Older age and the absence of flank pain were risk factors of AKI in our study, although the latter was not significant in multivariate analysis. The absence of flank pain may be associated with late diagnosis and treatment, leading to an unfavorable prognosis.

Previous studies have reported that patients with even mild AKI experience significantly higher mortality than those without AKI [29], and the role of AKI as an independent risk factor for mortality is well known [30–32]. According to one report, total mortality was 1.7% in patients with bilateral APN [19]. According to another report, overall mortality rate was 0.38% in 790 UTI patients [21]. However, these two studies did not describe the relationship between mortality and AKI. In our study, there was no significant difference in mortality between non-AKI and AKI groups (1.3% versus 0.79%) despite high incidence of AKI. It is possible that the total mortality in our study was so low (0.99%) that it did not affect the statistics between the two groups. Another lower mortality in AKI group might result from rapid recovering with general care, such as fluid replacement and adequate antibiotic therapy.

This study had some limitations. First, it had a retrospective design. As the research relied on medical records, it had a limited capacity to identify other possible causes of AKI such as nephrotoxic drugs and agents, which may have been used before admission to our hospital. Second, we used RIFLE classification for the diagnosis of AKI, but we only applied serum creatinine level and eGFR derived from the MDRD equation and could not classify patients based on urine output because the urine output of APN patients is not routinely recorded. Therefore, the incidence and severity of AKI may have been underestimated. Third, the research was conducted at a single center, so the results should not be generalized to the entire community. A well-designed and randomized prospective study is required to confirm our results. However, we believe that these limitations may be overcome by our large participant pool. Laboratory values were relatively well-recorded because patients were hospitalized, and identical laboratory conditions were applied to all patients. The same treatments were also performed on all patients.

## Conclusions

The incidence of AKI associated with APN was relatively higher (61.2%) in our study than in previous reports although most patients recovered from APN and AKI with general care, such as fluid replacement and adequate antibiotic therapy, excluding four patients who died (0.99%). No patients started renal replacement therapy and there was no case of progression to CKD in non-CKD groups. Our results also suggest that older age, complicated and bilateral APN, and initial shock are important risk factors associated with the occurrence of AKI. Severe inflammation represented by serum markers is also associated with AKI.

## Ethical approval

The study protocol was approved by the Institutional Review Board of Gyeongsang National University Hospital (IRB No.: 2016-01-014).

## References

[1] Hooton TM, Stamm WE Diagnosis and treatment of uncomplicated urinary tract infection. Infect Dis Clin North Am. 1997;11:551–581. 9378923 10.1016/s0891-5520(05)70373-1

[2] Ki M, Park T, Choi B, et al The epidemiology of acute pyelonephritis in South Korea, 1997–1999. Am J Epidemiol. 2004;160:985–993. 15522855 10.1093/aje/kwh308

[3] Ramakrishnan K, Scheid DC Diagnosis and management of acute pyelonephritis in adults. Am Fam Phys. 2005;71:933–942. 15768623

[4] Piccoli GB, Consiglio V, Deagostini MC, et al. The clinical and imaging presentation of acute "non complicated" pyelonephritis: a new profile for an ancient disease. BMC Nephrol. 2011;12:68. 22171968 10.1186/1471-2369-12-68 PMC3268718

[5] Akram AR, Singanayagam A, Choudhury G, et al. Incidence and prognostic implications of acute kidney injury on admission in patients with community-acquired pneumonia. Chest. 2010;138:825–832. 20435657 10.1378/chest.09-3071

[6] Hoste EAJ, Clermont G, Kersten A, et al. RIFLE criteria for acute kidney injury are associated with hospital mortality in critically ill patients: a cohort analysis. Crit Care. 2006;10:R73. 16696865 10.1186/cc4915 PMC1550961

[7] Ostermann M, Chang RWS Acute kidney injury in the intensive care unit according to RIFLE. Crit Care Med. 2007;35:1837–1843. 17581483 10.1097/01.CCM.0000277041.13090.0A

[8] Uchino S, Kellum JA, Bellomo R, et al. Beginning and Ending Supportive Therapy for the Kidney (BEST Kidney) Investigators: acute renal failure in critically ill patients: a multinational, multicenter study. JAMA. 2005;294:813–818. 16106006 10.1001/jama.294.7.813

[9] Vincent J-L, Sakr Y, Sprung CL, et al. Sepsis occurrence in acutely ill patients investigators: sepsis in European intensive care units: results of the SOAP study. Crit Care Med. 2006;34:344–353. 16424713 10.1097/01.ccm.0000194725.48928.3a

[10] Guerin C, Girard R, Selli JM, et al. Initial versus delayed acute renal failure in the intensive care unit. A multicenter prospective epidemiological study. Rhône-Alpes Area Study Group on Acute Renal Failure. Am J Respir Crit Care Med. 2000;161:872–879. 10712336 10.1164/ajrccm.161.3.9809066

[11] Hoste EAJ, Lameire NH, Vanholder RC, et al. Acute renal failure in patients with sepsis in a surgical ICU: predictive factors, incidence, comorbidity, and outcome. J Am Soc Nephrol. 2003;14:1022–1030. 12660337 10.1097/01.asn.0000059863.48590.e9

[12] Yegenaga I, Hoste E, Van Biesen W, et al. Clinical characteristics of patients developing ARF due to sepsis/systemic inflammatory response syndrome: results of a prospective study. Am J Kidney Dis. 2004;43:817–824. 15112172 10.1053/j.ajkd.2003.12.045

[13] Jones SR Acute renal failure in adults with uncomplicated acute pyelonephritis: case reports and review. Clin Infect Dis. 1992;14:243–246. 1571439 10.1093/clinids/14.1.243

[14] Söylemezoğlu O, Kale G, Saatçi U, et al. Acute renal failure due to acute pyelonephritis. Int Urol Nephrol. 1995;27:137–139. 7591568 10.1007/BF02551309

[15] Nahar A, Akom M, Hanes D, et al. Pyelonephritis and acute renal failure. Am J Med Sci. 2004;328:121–123. 15311172 10.1097/00000441-200408000-00009

[16] Sqalli TH, Hamzaoui H, Guiard E, et al. Severe renal failure in acute bacterial pyelonephritis: do not forget corticosteroids. Saudi J Kidney Dis Transpl. 2010;21:118–122. 20061705

[17] Sung SA, Kang YS, Lee SY, et al. Acute renal failure in acute pyelonephritis. Korean J Med. 2003;64:217–224.

[18] Jang SH, Lee CS, Lee MY, et al. Clinical differences in acute kidney injury between unilateral acute pyelonephritis and bilateral acute pyelonephritis. Korean J Med. 2012;82:696–703.

[19] Lee YJ, Cho S, Kim SR Unilateral and bilateral acute pyelonephritis: differences in clinical presentation, progress and outcome. Postgrad Med J. 2014;90:80–85. 24255118 10.1136/postgradmedj-2013-131935

[20] Bellomo R, Ronco C, Kellum JA, et al. Acute renal failure—definition, outcome measures, animal models, fluid therapy and information technology needs: the Second International Consensus Conference of the Acute Dialysis Quality Initiative (ADQI) Group. Crit Care. 2004;8:R204–R212. 15312219 10.1186/cc2872 PMC522841

[21] Hsiao CY, Yang HY, Hsiao MC, et al. Risk factors for development of acute kidney injury in patients with urinary tract infection. PLoS One. 2015;10:e0133835. 26213991 10.1371/journal.pone.0133835 PMC4516244

[22] Sakhuja A, Kumar G, Gupta S, et al. Acute kidney injury requiring dialysis in severe sepsis. Am J Respir Crit Care Med. 2015;192:951–957. 26120892 10.1164/rccm.201502-0329OC

[23] Kooman JP, Barendregt JN, van der Sande FM, et al. Acute pyelonephritis: a cause of acute renal failure? Neth J Med. 2000;57:185–189. 11063864 10.1016/s0300-2977(00)00063-2

[24] Yun SE, Jeon DH, Kim MJ, et al. The incidence, risk factors, and outcomes of acute kidney injury in patients with pyogenic liver abscesses. Clin Exp Nephrol. 2015;19:458–464. 25091457 10.1007/s10157-014-1016-8

[25] Hwang K, Jang HN, Lee TW, et al. Incidence, risk factors and clinical outcomes of acute kidney injury associated with scrub typhus: a retrospective study of 510 consecutive patients in South Korea (2001–2013). BMJ Open. 2017;15:e013882. 10.1136/bmjopen-2016-013882 PMC5353335 28298367

[26] Coca SG, Yusuf B, Shlipak MG, et al. Long-term risk of mortality and other adverse outcomes after acute kidney injury: a systematic review and meta-analysis. Am J Kidney Dis. 2009;53:961–973. 19346042 10.1053/j.ajkd.2008.11.034 PMC2726041

[27] Jones J, Holmen J, De Graauw J, et al. Association of complete recovery from acute kidney injury with incident CKD stage 3 and all-cause mortality. Am J Kidney Dis. 2012;60:402–408. 22541737 10.1053/j.ajkd.2012.03.014 PMC3422603

[28] Mammen C, Al Abbas A, Skippen P, et al. Long-term risk of CKD in children surviving episodes of acute kidney injury in the intensive care unit: a prospective cohort study. Am J Kidney Dis. 2012;59:523–530. 22206744 10.1053/j.ajkd.2011.10.048

[29] Chertow GM, Burdick E, Honour M, et al. Acute kidney injury, mortality, length of stay, and costs in hospitalized patients. J Am Soc Nephrol. 2005;16:3365–3370. 16177006 10.1681/ASN.2004090740

[30] Nisula S, Kaukonen K-M, Vaara ST, et al. Incidence, risk factors and 90-day mortality of patients with acute kidney injury in Finnish intensive care units: the FINNAKI study. Intensive Care Med. 2013;39:420–428. 23291734 10.1007/s00134-012-2796-5

[31] Joannidis M, Metnitz B, Bauer P, et al. Acute kidney injury in critically ill patients classified by AKIN versus RIFLE using the SAPS 3 database. Intensive Care Med. 2009;35:1692–1702. 19547955 10.1007/s00134-009-1530-4

[32] Barrantes F, Tian J, Vazquez R, et al. Acute kidney injury criteria predict outcomes of critically ill patients. Crit Care Med. 2008;36:1397–1403. 18434915 10.1097/CCM.0b013e318168fbe0

